# Robotic Partial Nephrectomy for Renal Tumors Larger than 4 cm: A Systematic Review and Meta-analysis

**DOI:** 10.1371/journal.pone.0075050

**Published:** 2013-10-08

**Authors:** Liangkuan Bi, Caixia Zhang, Kaiwen Li, Xinxiang Fan, Kewei Xu, Jinli Han, Hai Huang, Hao Liu, Wen Dong, Xiangyun Yang, Jian Huang, Tianxin Lin

**Affiliations:** 1 Department of Urology, Sun Yat-sen Memorial Hospital, Sun Yat-sen University, Guangzhou, China; 2 Key Laboratory of Malignant Tumor Gene Regulation and Target Therapy of Guangdong Higher Education Institutes, Sun Yat-sen University, Guangzhou, China; H. Lee Moffitt Cancer Center & Research Institute, United States of America

## Abstract

**Background:**

With the establishment of minimally invasive surgery in society, the robot has been increasingly widely used in the urologic field, including in partial nephrectomy. This study aimed to comprehensively summarize the currently available evidence on the feasibility and safety of robotic partial nephrectomy for renal tumors of >4 cm.

**Method and Findings:**

An electronic database search of PubMed, Scopus, Web of Science, and the Cochrane Library was performed. This systematic review and meta-analysis was based on all relevant studies that assessed robotic partial nephrectomy for renal tumors of >4 cm. Five studies were included. The meta-analysis involved 3 studies from 11 institutions including 154 patients, while the narrative review involved the remaining 2 studies from 5 institutions including 64 patients. In the meta-analysis, the mean ischemic time, operation time, and console time was 28, 319, and 189 minutes, respectively. The estimated blood loss and length of stay was 317 ml and 3.8 days, respectively. The rates of conversion, positive margins, intraoperative complications, postoperative complications, hilar clamping, and collecting system repair were 7.0%, 3.5%, 7.0%, 9.8%, 93.9%, and 47.5%, respectively. The narrative review showed results similar to those of the meta-analysis.

**Conclusions:**

Robotic partial nephrectomy is feasible and safe for renal tumors of >4 cm with an acceptable warm ischemic time, positive margin rate, conversion rate, complication rate, operation time, estimated blood loss, and length of stay.

## Introduction

Partial nephrectomy (PN) is the gold standard for treatment of small renal masses and selected T1b tumors for which removal is technically feasible [1]. Evolution has progressed from open radical nephrectomy (RN) through open PN to minimally invasive PN, including laparoscopic PN (LPN) and robotic PN (RPN) [2]. For small renal masses, RPN provides benefits similar to those provided by LPN with acceptable safety [3], [4].

PN for larger tumors (>4 cm) is reportedly similar to RN by a laparoscopic approach in terms of oncologic and functional outcomes [5], [6], providing evidence of the feasibility of this minimally invasive procedure. Since the first introduction of RPN in 2004 [7], renal tumors of >4 cm have reportedly been removed by this technique in some large intuitions [8], [9]. However, only a limited number of cases have been reported.

We performed a systematic review and meta-analysis of the available literature on RPN for renal tumors of >4 cm and herein discuss its feasibility and safety in terms of perioperative and early oncologic outcomes.

## Materials and Methods

A prospective protocol of objectives, literature-search strategies, inclusion and exclusion criteria, outcome measurements, and methods of statistical analysis was prepared *a priori* according to the Preferred Reporting Items for Systematic Reviews and Meta-Analyses [10].

### Literature-search Strategy

A literature search was performed using the electronic databases of PubMed, Web of Science, Scopus, and the Cochrane Library in April 2013. The following terms and their combinations were searched: *robot* or *robotic*, *partial* or *nephron-sparing*, *nephrectomy*, and *4 cm*. The Related Articles function was also used to broaden the search. Additional studies were manually searched in the reference lists of all retrieved articles. When multiple reports describing the same population were published, the most recent or complete report was used in the meta-analysis. However, it was not applicable if the outcome measures were mutually exclusive or measured in different time periods. The studies excluded from the meta-analysis underwent a narrative synthesis.

### Inclusion and Exclusion Criteria

All articles and meeting abstracts that reported the performance of RPN for renal tumors of >4 cm in all age groups and that had at least one of the quantitative outcomes mentioned in the next section of this paper were included.

### Data Extraction and Outcomes of Interest

Two authors (Li and Bi) independently extracted and summarized the data for the following parameters: authors, publication year, country, number of institutions, instruments for diagnosis, number of patients, tumor size, age, gender, body mass index, American Society of Anesthesiologists score, nephrometry score, and outcomes of interest. Any disagreement was resolved by the adjudicating senior authors (Huang and Lin).

The primary outcomes were warm ischemic time, conversion rate, positive margin rate, and complication rate. The secondary outcomes were operation room time, console time, estimate blood loss, hilar clamping rate, collecting system repair rate, blood transfusion rate, and length of stay.

### Statistical Analysis

The meta-analysis was performed using Meta-Analyst [11]. The DerSimonian and Laird random method was used to combine dichotomous variables to rates. Continuous variables were combined to weighted mean with a random method. For studies that presented continuous data as medians and ranges, the means and standard deviations were calculated using statistical algorithms described by Hozo et al [12]. Statistical heterogeneity between studies was assessed using the chi-square test with significance set at *p<*0.10, and heterogeneity was quantified using the *I^2^* statistic with significance set at *I^2^*>50% [13]. The use of Egger’s funnel plots was initially planned, but were eventually not used to assess the possibility of publication bias because of either the limited number of studies included for the meta-analysis or the significant heterogeneity among studies [14]. Studies not used for the meta-analysis were reviewed and underwent a narrative synthesis.

## Results

### Literature Search and Study Characteristics

**Figure 1:**
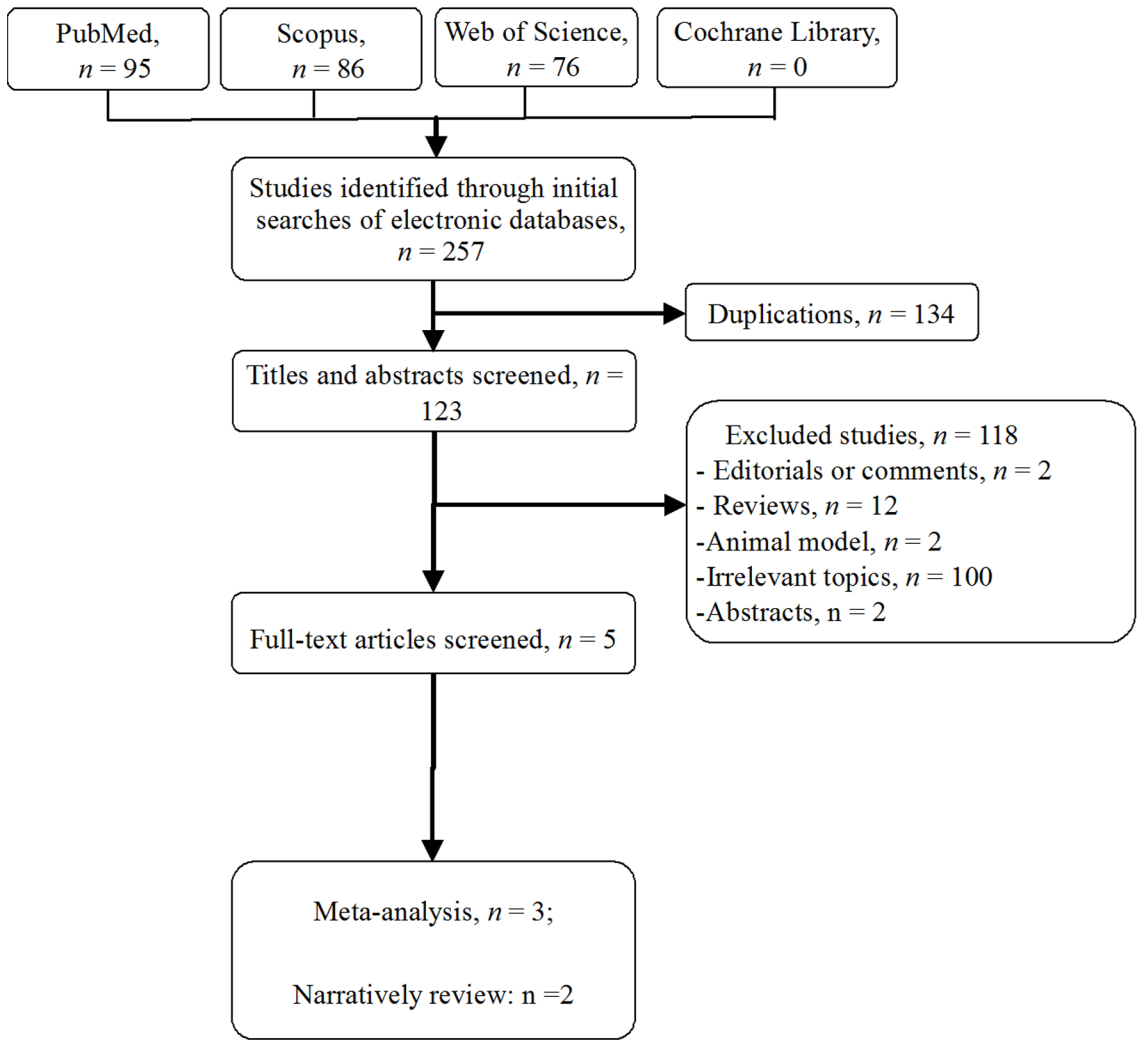
Flow diagram of studies identified, included, and excluded.

Five studies [8], [9], [15]–[17] fulfilled the predefined inclusion criteria and were included in the final analysis (Fig. 1). Table 1 shows the characteristics of the included studies. Two studies [9], [17] may have had some overlapping data as reported by Petros et al [16]. They were reviewed by a narrative synthesis. The other 3 studies [8], [15], [16] from 11 institutions including 154 patients were included in the meta-analysis.

**Table 1:** Characteristics of the studies included.

First Author, Yearof Publication	Country	Study Design	Institutions, no.	Instrument for Diagnosis	Patients, no.	Age, years, Mean(Standard Deviation)/Median(Range)	Male, no.	Nephrometry RENAL Score, Mean(Standard Deviation)/Median(Range)	Clinical Tumor Size, Median (Range), cm
**Petros2012 ** [**16**]	USA	R	4	Radiography	83	61 (12)	52	8.0 (4–11)	5.0(4.1–11)
**Lecomte2013 ** [**8**]	France	P	6	US+ CT	54	62 (31–81)	35	7.0 (1.5)	4.5(4–7)*
**Gupta2013 ** [**15**]	USA	R	1	Radiography	17/19¶	47 (26–76)	9	8.9 (1.3)	5.0(4.1–15)
**Ficarra2012 ** [**9**]	USA+ Italy	R	4	MR or CT	49	60 (52–66)#	NA	10 (8–10)#, †	5.0(4.4–5.5)#
**Patel2010 ** [**17**]	USA	P	1	MR or CT	15	59 (44–76)	9	NA	5.0(4.1–7.9)

P = prospectively collected data; R = retrospectively; US = ultrasonography; CT = computerized tomography; MR = magnetic resonance; NA = data not available;

* pathological tumor size;

# median (interquartile range);

¶ 17 patients with 19 operations;

† PAUDA score.

### Primary Outcomes

#### Warm ischemic time

The median warm ischemic time was 22 to 36 minutes [8], [9], [15]–[17]. Pooling of the data of 11 institutions [8], [15], [16] showed a mean of 28 minutes (95% confidence interval [CI], 21–34 minutes) (Table 2).

**Table 2:** Perioperative information in meta-analysis.

Variables	No. of Institutions		No. of Procedures	Mean	95% Confidence Interval
**Primary Outcomes**					
Warm ischemic time, min	11		153	28	21–34
Conversion#, %	11		156	7.0	2.6–17.7
Conventional laparoscopic PN				2.9	1.2–7.2
Open PN				4.7	0.9–21
Positive margin, %	11		153	3.5	1.1–10.5
Intraoperative complication#, %	11		156	7.0	2.6–17.7
Postoperative complication, %	11		153	9.8	4.3–20.8
Major complication*, %	11		153	4.7	2.3–9.5
**Secondary Outcomes**					
Operation room time, min	5		70	319	193–445
Console time, min	10		137	189	176–202
Estimate blood loss, ml	11		153	317	43–591
Hilar clamping, %	11		153	93.9	88.7–96.8
Collecting system repair, %	7		99	47.5	37.9–57.3
Length of stay, days	10		137	3.8	1.9–5.7

PN = partial nephrectomy;

* Clavien-Dino classification grade >3;

# Conversion was treated as intraoperative complications according to Clavien-Dino classification.

#### Conversion

No conversion was reported by Petal et al [17] or Ficarra et al [9] (Table 3). However, the combined conversion rate of 11 institutions [8], [15], [16] as estimated by the random-effects model was 7.0% (95% CI, 2.6%–17.7%). Reported conversions were grouped into conventional laparoscopic PN and open PN with estimated rates of 2.9% and 4.7% (95% CI, 1.2%–7.2% and 0.9%–21%), respectively (Table 2).

**Table 3:** Comparison between meta-analysis and narrative review.

Variables	Petal 2010 [17]	Ficarra2012 [9]	Meta-analysis
**Cases, N**	15	49	156
**Median warm ischemic time, min (IQR)**	25 (20–30)	22 (18–28)	28(21–34)*
**Conversion, %**	0	0	7.0
**Positive margin, %**	0	5.1	3.5
**Intraoperative complication, %**	0	4	7.0
**Postoperative complication, %**	26.6	26.5	9.8
** Major complication** † **, %**	19.8	8	4.7
**Median operation room time, min (IQR)**	275 (229–344)	177 (138–200)	319(193–445)*
**Median console time, min (IQR)**	NA	145 (112–177)	189(176–202)*
**Median estimated blood loss, ml (IQR)**	100 (75–200)	120 (62–237)	317(43–591)*
**Hilar clamping, %**	86.7	NA	93.9
**Collecting system repair, %**	71	57	47.5
**Median length of stay, days (IQR)**	2 (2–4)	NA	3.8(1.9–5.7)*

NA = data not available; IQR = interquartile range;

* mean(95% confidence interval);

† Clavien-Dino classification grade >3.

#### Positive margin

The positive margin rate was reported as 5.1% by Ficarra et al [9] and 0% by Petal et al [17] (Table 3). Pooling of the data of 11 institutions [7], [14], [15] indicated a rate of 3.5% (95% CI, 1.1%–10.5%) (Table 2).

#### Complications

The intraoperative complication rate was reported as 4% by Ficarra et al [9] and 0% by Petal et al [17] (Table 3). No intraoperative complications were declared among the 11 institutions [7], [14], [15]. However, conversions were treated as complications according to the Clavien-Dindo classification. The estimated rate was 7.0% (95% CI, 2.6%–17.7%) (Table 2).

The postoperative complication rate was reported as 26.5% by Ficarra et al [9] and 26.6% by Petal et al [17] (Table 3). Nonetheless, the combined rate from 11 institutions [7], [14], [15] was lower at 9.8% (95% CI, 4.3%–20.8%) (Table 3). The major complication rate was reported as 8% by Ficarra et al [9] and 19.8% by Petal et al [17] (Table 3). The combined major complication rate [7], [14], [15] was 4.7% (95% CI, 2.3%–9.5%) (Table 2). All reported major complications necessitating intervention were urine leakage and bleeding/pseudoaneurysm. One and two cases of urine leakage were reported by two [8], [15] and the remaining three studies [9], [16], [17], respectively. One and two cases of bleeding/pseudoaneurysm were reported by two [16], [17] and one study [8], respectively.

### Secondary Outcomes

#### Operative room time and console time

The median operative room and console times were 177 to 275 minutes [9], [17] and 145 minutes [9], respectively (Table 3). Pooling of the operative room time data from six institutions [8], [15] showed a mean of 319 minutes (95% CI, 193–445 minutes), and console time data from 10 institutions [8], [16] showed a mean of 189 minutes (95% CI, 176–202 minutes) (Table 2).

#### Estimated blood loss and length of stay

The median estimated blood loss was 100 to 120 ml [9], [17]. The combined data from 11 institutions [8], [15], [16] showed a mean estimated blood loss of 317 ml (95% CI, 43–591 ml). The median length of stay was 2 days as reported by Petal et al [17]. The combined data from 10 institutions [8], [16] showed a mean length of stay of 3.8 days (95% CI, 1.9–5.7 days) (Tables 2 and 3).

#### Hilar clamping and collecting system repair

The hilar clamping rate was 86.7% as reported by Petal et al [17]. The combined data from 11 institutions [8], [15], [16] showed a rate of 93.9% (95% CI, 88.7%–96.8%). The collecting system repair rate was 57% to 71% [9], [17]. The combined data from seven institutions [15], [16] showed a repair rate of 47.5% (95% CI, 37.9%–57.3%) (Tables 2 and 3).

## Discussion

The present systematic review provided a comprehensive overview of the current evidence on the feasibility and safety of RPN for renal tumors of >4 cm. It showed an acceptable warm ischemic time, conversion rate, complication rate, operation time, estimated blood loss, and length of stay.

In the treatment of renal masses of <4 cm, LPN as a minimally invasive technique has significantly evolved to the point at which the short- and long-term safety rivals that of open PN [18], [19]. RPN was recently introduced as a feasible and safe alternative to LPN in terms of perioperative outcomes. A meta-analysis comparing RPN with LPN for T1a tumors concluded that there exists no significant difference in perioperative variables between the two techniques [4]. Another meta-analysis of the treatment of tumors with a mean size of <4 cm indicated that RPN may be more suitable than LPN in terms of decreased warm ischemic times [3]. However, detailed comparisons of long-term outcomes should be performed.

The treatment of renal tumors of >4 cm may be complicated. However, some studies have been dedicated to the demonstration of the feasibility of LPN for large tumors. In a comparison of laparoscopic PN with laparoscopic RN, the overall mortality, cancer-specific mortality, and recurrence rates were equivalent [6]. With median follow-up periods of 15 and 21 months for the laparoscopic PN and RN cohorts, Deklaj et al indicated that LPN for T1b renal tumors provides superior preservation of renal function [5]. All of these intermediate-term data support the possibility of wide application of LPN to large renal tumors in future studies [5], [6].

LPN has been successfully applied to selected renal tumors of >4 cm for which removal is technically feasible [20]–[24]. Table 4 compares data reported in previous studies of LPN [20]–[24] and data analyzed in the present meta-analysis. A prolonged warm ischemic time, higher risk of perioperative complications, and higher rate of positive surgical margins may represent the most important concerns potentially limiting the diffusion of RPN for renal tumors of >4 cm. In this meta-analysis, the pooled mean warm ischemic time of RPN was 28 minutes, which is comparable with that of LPN (21.9–38 minutes) [20]–[24]. However, the rates of positive margins and postoperative complications were 3.5% and 9.8%, respectively. These rates seemed lower than those of LPN (3.8%–6.5% and 24%–42%, respectively) [20]–[24]. All other outcomes were acceptable (Table 4). Given the well-established technique and widespread application of LPN, RPN may be a feasible alternative to LPN for large renal tumors.

**Table 4:** Comparison between meta-analysis and other minimally invasive studies on partial nephrectomy for renal tumors >4 cm.

Variables	Rais2008 [21]	Eng2009[22]	Simmons2009[23]	Porpiglia2010[24]	Sprenkle2012[20]	Meta-analysis
**Procedure**	Laparoscopic	Laparoscopic	Laparoscopic	Laparoscopic	Robotic andLaparoscopic	Robotic
**Cases, N**	34	26	58	63/41¶	54	156
**Mean warm ischemic time, min (SD)**	21.9(13.7)	30.3 (10.9)	38 (11.9)	25.7 (8.3)	37 (31–41)#	28(21–34)*
**Conversion, %**	NA	15	4	7.3	13	7.0
**Positive margin, %**	5.3	3.8	6.5	6.5	4	3.5
**Intraoperative complication, %**	4.0	NA	7	7.3	19	7.0
**Postoperative complication, %**	37.0	42	24	26	33	9.8
** Major complication** † **, %**	NA	15	8.6	14.6	15	4.7
**Mean operation room time, min (SD)**	NA	NA	NA	NA	NA	319(138–200)*
**Mean console time, min (SD)**	199.2(57.2)	234(111)	228 (78)	154 (62)	NA	189(176–202)*
**Mean estimated blood loss, ml (SD)**	406.3(354.3)	247(252)	284 (302)	230 (143)	300 (144–438)#	317(43–591)*
**Hilar clamping, %**	NA	NA	NA	100	93	93.9
**Collecting system repair, %**	NA	88.5	90	43	NA	47.5
**Mean length of stay, day (SD)**	4.1(2.7)	NA	3.5 (1.5)	7 (3.5)	3 (2–5)#	3.8(1.9–5.7)*

NA = data not available; SD = standard deviation;

¶ Complete data on complications were available for 41 patients;

* mean(95% confidence interval);

# median(interquartile range);

† Clavien-Dino classification grade >3.

The most important finding in this study is that the success of LPN can be rapidly transited to RPN. Lavery et al [25] focused on one experienced surgeon and highlighted the quick learning curve associated with the transition from LPN to RPN. There were no significant differences in warm ischemic time, estimated blood loss, or length of hospital stay when comparing the first 20 RPN and the last 18 LPN procedures. RPN achieves an operation time similar to that of LPN after five procedures. Similarly, Pierorazio et al [26] concluded that the transition from LPN to RPN can be undertaken without an additional learning curve and is associated with immediate benefits after approximately 25 LPN procedures. When performed by a surgeon with extensive robotic experience, RPN has a short learning curve to reach a warm ischemic time of <20 min, console time of <100 min, limited blood loss, and an acceptable overall complication rate [27]. Kaouk et al [28] showed that once past the learning curve, a significantly decreased estimated blood loss, transfusion rate, conversion rate, complication rate, operative time, and length of stay was obtained in the largest reported series comparing early and later experiences of RPN. Nevertheless, further high-quality studies are needed to determine whether the learning curve of RPN for renal tumors of >4 cm can be easily passed.

Despite the feasibility of RPN, cost might be an important factor impacting the choice of operation procedure. Mir et al [29] compared the costs of PN carried out by laparoscopic and robotic procedures. They performed a systematic review and meta-analysis and indicated that RPN is associated with higher costs than LPN because of maintenance and instrumentation. Yu et al [27] found that robotic surgery costs significantly more than laparoscopic procedures, although it is associated with a significantly shorter hospital stay, fewer complications, and a lower transfusion rate. However, there has been no social cost analysis of factors involved in quicker recovery and shorter convalescence by robotic procedures in urologic surgery [30]. It is estimated that the total costs of RPN are about $1600 more per person [29], [30]. A significant decrease in robotic costs is required for RPN to be cost-effective.

The present systematic review and meta-analysis has some limitations that must be considered. The main limitation is that it relied on a minority of eligible studies. Only five studies were included, and just three of them [8], [15], [16] were used for the meta-analysis. Because of some potentially overlapping data, the other two studies were reviewed narratively. The sample size of some studies was small, limiting the statistical power. In addition, because most studies originated from high-volume institutions or centers of excellence, the results may be difficult to transfer to community-based practice. Finally, the follow-up period was generally short, so long-term outcomes remained to be evaluated.

However, the procedure of RPN for renal tumors of >4 cm has only been applied for a short period of time, in limited institutions, and in small sample sizes. This present meta-analysis with 11 institutions including 153 patients and narrative review of 5 institutions including 64 patients may provide better evidence for the feasibility of RPN for renal tumors of >4 cm.

## Conclusions

This systematic review and meta-analysis indicates that RPN is feasible and safe for renal tumors of >4 cm with an acceptable warm ischemic time, conversion rate, complication rate, operation time, estimated blood loss, and length of stay. Nevertheless, future large-volume, well-designed prospective and randomized studies comparing PN for renal tumors of >4 cm by robotic, laparoscopic, or open procedures and that compare PN and RN for renal tumors of >4 cm by robotic procedures are needed to confirm and update the findings of this analysis.

## Supporting Information

Checklist S1 **PRISMA 2009 Checklist.**
(DOC)

Text S1 **Take Home Message.**
(DOC)
