# Supplementary material for: Robotic Partial Nephrectomy for Renal Tumors Larger than 4 cm: A Systematic Review and Meta-analysis
Source: PLoS One. 2013 Oct 8;8(10):e75050. doi: 10.1371/journal.pone.0075050 (PMC3792962; doi:10.1371/journal.pone.0075050)
Supplement: Text S1 — Take Home Message. (DOC) [file pone.0075050.s002.doc]

**Take Home Message**

This is a systematic review and meta-analysis indicates robot partial nephrectomy to be feasible and safe for renal tumors of >4cm with acceptable warm ischemic time, conversion rates, complication rates, operation time, estimated blood loss, and length of stay.
